# Gradient Fractionated Separation of Chondrogenically Committed Cells Derived from Human Embryonic Stem Cells

**DOI:** 10.1089/biores.2014.0051

**Published:** 2015-01-01

**Authors:** Natasja Leth Joergensen, Anette Gabrielsen, Martin Lind, Helle Lysdahl

**Affiliations:** ^1^ Orthopaedic Research Laboratory, Aarhus University Hospital, Aarhus, Denmark.; ^2^ Ciconia Aarhus Private Hospital, Aarhus, Denmark.; ^3^ Sports Trauma Clinic, Aarhus University Hospital, Aarhus, Denmark.

**Keywords:** Human embryonic stem cells, micromass culture, gradient fractionation, chondrogenic commitment

## Abstract

Cartilage regeneration is a fast growing field that combines biotechnology and molecular techniques in creating new tissue mimicking the native microenvironment. Human embryonic stem cells (hESCs) are a highly potent cell source for cartilage regeneration owing to their infinite proliferation capacity and pluripotency. Thus, lineage-specific differentiation of hESCs often results in populations with cellular heterogeneity. Chondrogenesis was induced through high-density micromass culture of hESCs and by addition of chondrogenic medium; 1:100 ITS^+^, 100 nM dexamethasone, 40 μg/ml l-proline, 50 μg/mL ascorbic acid-2-phosphate, 1:100 Knockout serum, and 10 ng/mL TGFβ3. At day 14 micromasses were dissociated and chondrogenically committed cell separated in a fraction-based discontinuous density gradient. After fractionation the chondrogenically committed cells were analyzed with regard to embryonic- and chondrogenic gene expression and fraction F3 and F4 with histology. In general, we found that the chondrogenic condition compared with the control condition had a significant effect on the following gene expression levels: *NANOG*, *OCT4*, *SOX5*, *SOX9*, *ACAN*, and *COL2A1* in all fractions. Furthermore, we found in the chondrogenic condition that *NANOG*, *OCT4*, and *SOX9* were significantly higher in F4 compared with F3, whereas *COL2A1* and the ratio *COL2A1:COL1A1* were significantly lower. Additionally, toluidine blue pH 4 stains of pellet cultures of F3 and F4 revealed that cells from F3 were more homogenous in morphology than F4. In conclusion, we propose a simple strategy to obtain more homogenous population of chondrogenically committed cells from hESCs using micromass culture and discontinuous density gradient separation.

## Introduction

Articular cartilage regeneration represents one of the major challenges in orthopedic surgery. Even though autologous articular chondrocytes have the potential to produce various degrees of hyaline cartilage *in vivo* the cells are very limited in availability.^1^ This facilitates the interest of using human embryonic stem cells (hESCs) for cartilage regeneration owing to their infinite proliferation capacity and pluripotency. Several studies report that lineage commitment of hESCs in a certain direction often results in low differentiation efficiency and cellular heterogeneity in the differentiated cell population, which poses the risk of tumorigenicity *in vivo.*^2^ Previously, the fate decision of hESCs has been induced by the use a high-density micromass system for chondrogenic commitment.^3–5^

High-density micromass is a cell culturing method that more closely simulates condensation in chondrogenesis *in vivo* by balancing the optimal cell density environment and allowing cell–cell contact, and supplies the proper diffusion of nutrients;^3,6^ however, the micromass culture does not entirely allow uniform lineage commitment. Some cells will enter into chondrogenic stages, while others do not undergo overt differentiation from stem cells. Therefore, the purity of cells is of tremendous interest in order to establish desirable population of cells for research and clinical application. Recent studies have suggested a simple alternative for cell separation in stem cell research using discontinuous density gradients.^7–9^ This method has been used for years to isolate cells of different densities, for instance, isolation of bone marrow progenitor cells and human sperm cell enrichment.^10–12^ The aim of this study was to obtain a homogenous chondrogenically committed cell population derived from hESCs. We hypothesized that this could be accomplished by combining the micromass culture technique following fraction separation using discontinuous density gradient.

## Materials and Methods

### Micromass culture

Cell line hESCs CLS1 (male) was approved by the Central Denmark Region Committees on Biomedical Research Ethics (J. No. 20090205). Culturing hESCs was performed as previously described.^13^ hESCs were passaged by trypsinization and directly plated in 10 μL droplet high-density micromasses of 25×10^6^ cell/mL in 24-well tissue culture plates and cultured up to 14 days in either the control condition or the chondrogenic condition. Control medium consisted of Dulbecco's modified Eagle's medium (DMEM)/F12 (21063, Gibco, Life technologies) supplemented with 1:10 fetal bovine serum (10270, Gibco) and 1:10 knockout serum replacement (KSR) (10828-028, Gibco). Chondrogenic medium consisted of DMEM/F12 supplemented with 1:100 ITS™^+^ Premix (354352, BD Bioscience), 1:100 KSR, 40 μg/mL L-proline (P0380, Sigma-Aldrich), 50 μg/mL ascorbic acid 2-phosphate (A8960, Sigma-Aldrich), 100 nM dexamethasone (D2915, Sigma-Aldrich), and 10 ng/mL recombinant human TGFβ3 (243-B3, R&D Systems).

### Discontinuous density gradient

Micromasses were enzymatically dissociated using 1.5 mg/mL collagenase (C8176, Sigma) and 0.01 g/mL collagenase type II (17101-015, Life Technologies) in DMEM/F12 and separated in a discontinuous density gradient prepared from a 100% Suprasperm stock (10970500A, ORIGIO). Suprasperm (100%) was diluted with DMEM/F12 to the gradient concentrations of 10%, 30%, 35%, and 40%, respectively. Each of the solutions was gently mixed to ensure homogeneity. Layering of the gradient was performed by gently stacking 1 mL per density fraction from top till bottom in the following order 10%, 30%, 35%, and 40%. The dissociated cells were suspended in 1 mL DMEM/F12 and gently layered upon the 10% density fraction. The discontinuous density gradient was centrifuged once at 50 *g* for 15 min followed by 1000 *g* for 15 min. Fraction bands appeared at the interface between: F1: 0/10%; F2: 10/30%; F3; 30/35%; and F4: 35/40%.

### Total RNA extraction and quantitative polymerase chain reaction

Total RNA was extracted from cells within the four fractions F1: 0/10%; F2: 10/30%; F3; 30/35%; and F4: 35/40% using the GenElute^™^ Mammalian Total RNA Miniprep Kit (RTN 350, Sigma-Aldrich) according to the procedure of the manufacturer. The RNA concentration, purity, integrity, DNase 1 treatment, complementary DNA synthesis, and real-time quantitative PCR were performed as previously described.^14^ The following TaqMan^®^ Gene Expression Assays (4331182, Applied Biosystems) were used: Nanog homeobox; Hs02387400_g1(*NANOG*), POU class 5 homebox (*POU5F1*) (*OCT4*); Hs03005111_g1, sex determining region Y box 9 (*SOX9*); Hs00165814_m1, (*SOX6*); Hs00264525_m1, (*SOX5*); Hs00753050_s1, collagen type 2 alpha 1 (*COL2A1*); Hs00264051_m1, collagen type 1 alpha 1 (*COL1A1*); Hs00164004_m1, collagen type (*COL10*); Hs00166657_m1, Aggrecan (*ACAN*); and Hs00153936_m1. Data analysis was performed using 7500 Fast System Sequence Detection Software version 3.1. based on BestKeeper algorithm, values were normalized to the housekeeping genes CDC14A and RPl15.

### Pellet culture and histology

Fractionated cells were expanded in monolayer prior to pellet culture. At 80% confluency cells were trypsinized, pellets were formed (2.5×10^5^ cells/pellet) and cultured in chondrogenic medium. At day 28 pellets were fixed in 70% ethanol, infiltrated, and embedded in Technovit® 7100 (Heraeus Kulzer GmbH) according to the procedure of the manufacturer's instructions. Seven-micrometer sections were prepared and stained with toluidine blue 0.05% pH 4, washed in 99% ethanol, and visualized by light microscopy and software (NewCast version 3.0.9.0, Visiopharm).

### Statistics

Gene expression data was analyzed using analysis of variance. If interactions were observed the individual variables were analyzed separately. P-values lower than 0.05 were considered statistical significant. Stata Statistical Software: release 13.1 (StataCorp LP) was used for the statistical analyses.

## Results and Discussion

**FIG. 1.**
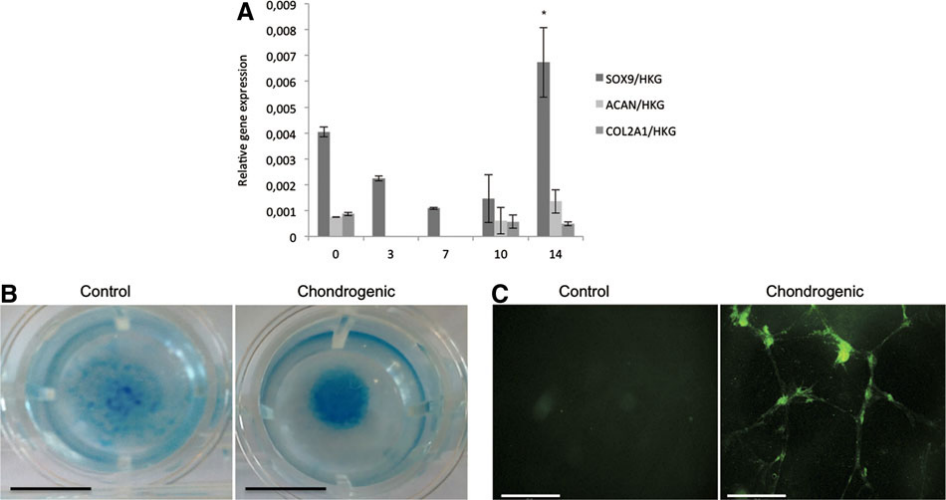
Chondrogenic differentiation of human embryonic stem cells. **(A)** Gene expression of *SOX9*, *ACAN*, and *COL2A1* after 0, 3, 7, 10, and 14 days of micromass culture with chondrogenic medium. Vertical axis represents the relative gene expression level. Horizontal axis represents the different time points (days) that micromasses where cultured with chondrogenic medium. Data are expressed as mean±standard deviation (*n*=2). *Significant difference between the time points (*p*<0.05). **(B)** Alcian blue pH 2.6 staining of proteoglycans and glycosaminoglycans in micromasses culture for 14 days in control medium or chondrogenic medium. Scale bar=0.8 cm. **(C)** Immunofluorescence anticollagen type 2 staining of micromasses cultured for 14 days in control medium or chondrogenic medium. Scale bar=850 μm.

Gene expression analysis revealed that *SOX9* was significantly increased in micromasses cultured for 14 days and that *ACAN* and *COL2A1* did not significantly change over time (Fig. 1A). Stains of micromasses cultured for 14 days showed that chondrogenic medium increased the deposition of proteoglycans and glycosaminoglycans and the presence of COL2 (Fig. 1B and 1C respectively). These results indicated that micromasses cultured in medium for 14 days contained chondrogenically committed cells able to express and synthesis chondrocyte specific markers. Therefore, micromasses cultured for 14 days were dissociated and fractionated in order to derive chondrogenically committed cells based on density. For that purpose, we designed a discontinuous density gradient consisting of four density fractions (F1: 0/10%; F2: 10/30%; F3: 30/35%; and F4: 35/40%).^7^

**FIG. 2.**
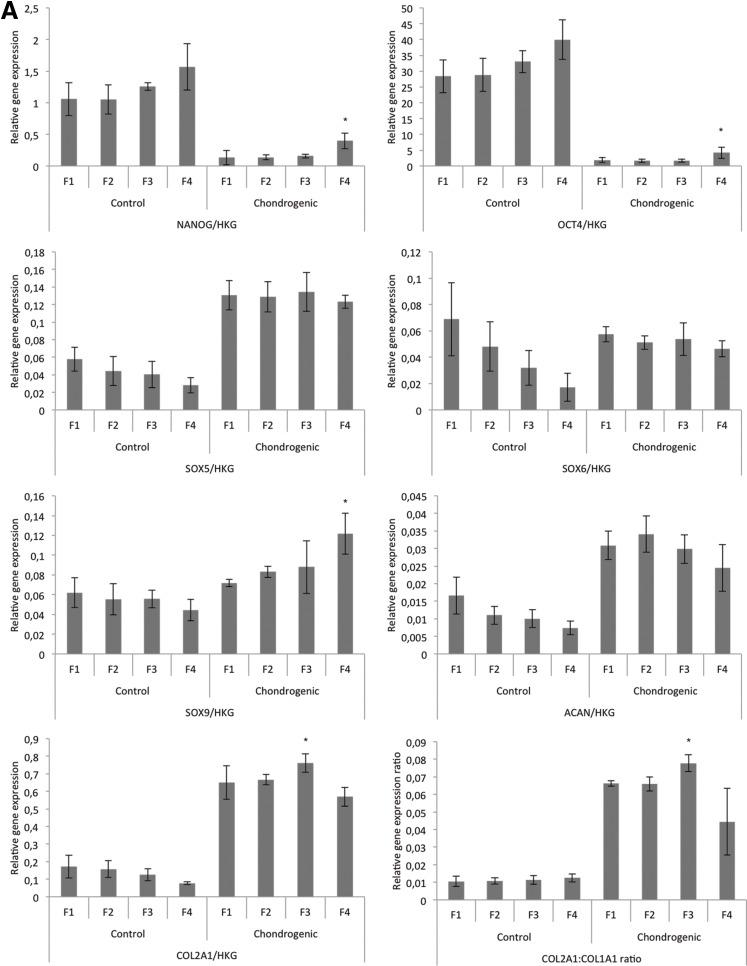

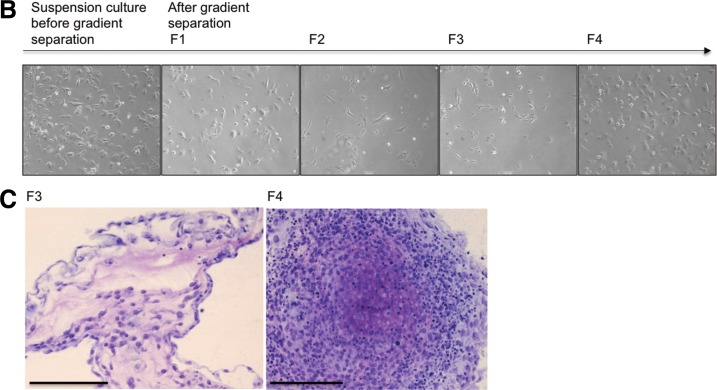
Analysis of the fractionated cell populations. **(A)** Relative gene expression of *OCT4, NANOG, SOX5, SOX6, SOX9, ACAN, COL2A1*, and *COL2A1:COL1A1* ratio in all four fractions (F1–F4) after density gradient separation. Vertical axes represent the relative gene expression level. Horizontal axes represent the four fractions (F1–F4) from the discontinuous density gradient for both control- and chondrogenic media. Data are expressed as mean±standard deviation (*n*=4). *Significant difference between fractions within the chondrogenic medium (*p*<0.05). **(B)** Monolayer culture of the cellular outcome before gradient separation and the 4 fractionated (F1–F4) cell populations with chondrogenic medium. **(C)** Histological sections of pelleted cells (F3 and F4) with chondrogenic medium after 28 days visualized by toluidine blue pH 4. Scale bar=150 μm.

After fractionating, gene expression of embryonic and chondrogenic markers was analyzed. Compared with the control condition, chondrogenic medium significantly reduced the expression of *NANOG* and *OCT4* and increased the expression of *SOX5*, *SOX9*, *ACAN*, and *COL2A1* in all fractions (Fig. 2A). At chondrogenic conditions F1, F2, and F3 had similar gene expression levels for all the genes analyzed. In F4 *NANOG*, *OCT4*, and *SOX9* were significantly higher in F4 compared with F3, whereas *COL2A1* and the ratio *COL2A1:COL1A1* were significantly lower (Fig. 2A). It was not possible to detect *COL10* in any of the fractions. The number of cell varied between the fractions. Visualization of the monolayer cultures indicated that F4 contained more cells than the other fractions (Fig. 2B). Toluidine blue pH 4 stains of pellet cultures of F3 and F4 revealed that cells from F3 were more homogenous in morphology than F4. Both fractions stained positive for proteoglycans (Fig. 2C).

This study demonstrates the derivation of a more homogenous chondrogenically committed cell population from hESCs using density gradient separation. Previous studies have used embryoid bodies, sorting hESCs for mesenchymal markers, coculture, or micromass culture in order to derive chondrogenically committed cells.^3,5,15–18^ In the present study the cell population derived from F3 contained cells with decreased embryonic markers, increased chondrogenic markers, and similar cell morphology. The study demonstrates that some cells have committed into chondrogenic direction while others have not undergone overt differentiation from stem cells. One of the major challenges using hESCs for repair of defective tissue is obtaining homogenous cell populations since a heterogeneous population of cells lead to teratoma formation.^19^ This study cannot exclude the problem of heterogeneity and *in vivo* teratoma formation. However, the presented method might represent simple means to obtain more homogeneous chondrogenically committed cell populations.

## Conclusion

We propose a simple strategy to fractionate hESCs that were driven into chondrogenesis. We obtain a more homogenous population of chondrogenically committed cells from hESCs using micromass culture and discontinuous density gradient separation. This strategy facilitates using hESCs for cartilageous regenerative medicine, since it potentially may reduce the risk of teratoma formation.

## Abbreviations Used

DMEM: Dulbecco's modified Eagle's medium
hESC: human embryonic stem cells
KSR: knockout serum replacement
